# All-In-Focus Polarimetric Imaging Based on an Integrated Plenoptic Camera with a Key Electrically Tunable LC Device

**DOI:** 10.3390/mi13020192

**Published:** 2022-01-26

**Authors:** Mingce Chen, Zhexun Li, Mao Ye, Taige Liu, Chai Hu, Jiashuo Shi, Kewei Liu, Zhe Wang, Xinyu Zhang

**Affiliations:** 1 National Key Laboratory of Science & Technology on Multispectral Information Processing, Huazhong University of Science & Technology, Wuhan 430074, China; D201780651@hust.edu.cn (M.C.); lzx98@hust.edu.cn (Z.L.); D202180975@hust.edu.cn (M.Y.); taige_liu@hust.edu.cn (T.L.); D201880681@hust.edu.cn (C.H.); D201980727@hust.edu.cn (J.S.); D202080878@hust.edu.cn (K.L.); M202072914@hust.edu.cn (Z.W.); 2 School of Artificial Intelligence and Automation, Huazhong University of Science & Technology, Wuhan 430074, China; 3 Innovation Insititute, Huazhong University of Science and Technology, Wuhan 430074, China

**Keywords:** liquid-crystal (LC) device, plenoptic camera, polarimetric imaging, visibility enhancement

## Abstract

In this paper, a prototyped plenoptic camera based on a key electrically tunable liquid-crystal (LC) device for all-in-focus polarimetric imaging is proposed. By using computer numerical control machining and 3D printing, the proposed imaging architecture can be integrated into a hand-held prototyped plenoptic camera so as to greatly improve the applicability for outdoor imaging measurements. Compared with previous square-period liquid-crystal microlens arrays (LCMLA), the utilized hexagonal-period LCMLA has remarkably increased the light utilization rate by ~15%. Experiments demonstrate that the proposed imaging approach can simultaneously realize both the plenoptic and polarimetric imaging without any macroscopic moving parts. With the depth-based rendering method, both the all-in-focus images and the all-in-focus degree of linear polarization (DoLP) images can be obtained efficiently. Due to the large depth-of-field advantage of plenoptic cameras, the proposed camera enables polarimetric imaging in a larger depth range than conventional 2D polarimetric cameras. Currently, the raw light field images with three polarization states including *I*_0_ and *I*_60_ and *I*_120_ can be captured by the proposed imaging architecture, with a switching time of several tens of milliseconds. Some local patterns which are selected as interested target features can be effectively suppressed or obviously enhanced by switching the polarization state mentioned. According to experiments, the visibility in scattering medium can also be apparently improved. It can be expected that the proposed polarimetric imaging approach will exhibit an excellent development potential.

## 1. Introduction

To describe the transmission characteristics of light waves in 3D space, fully 3D or “integral” photography was first introduced by Lippmann [1]. A similar concept of light fields was firstly proposed by Gershun in 1939 [2]. In 1991, Adelson and Bergen proposed a 7D function to represent the spatial distribution of geometric light beams, which was called the plenoptic function [3]. McMillan and Bishop discussed the representation of 5D light fields as a set of panoramic images at different 3D locations in 1995 [4]. Then, Levoy and Hanraham proposed a light field rendering theory in 1996 [5]. In the case of neglecting attenuation of light waves during transmission, the 5D plenoptic function can be simplified to 4D, which can also be parametrically characterized with two parallel planes. Based on the basic light field rendering methods, Ng et al. built the first hand-held plenoptic camera by directly inserting a microlens array between the main lens system and the imaging sensors in 2005, which was already called the standard plenoptic camera [6]. In 2009, Lumsdaine and Georgiev further proposed a kind of focused plenoptic camera, which can be used to obtain a much higher spatial resolution of the rendering images [7]. In general, the developed plenoptic camera can be utilized to easily record 4D plenoptic function, and thereby enable many unique applications, such as digital refocusing, multi-view imaging, depth estimation, and 3D target reconstruction. In particular, compared with conventional cameras, plentopic cameras have a larger depth of field [6,7,8].

To date, most of the developed plenoptic cameras capture only the geometric aspects of the plenoptic function, whereas other key physical parameters of the imaging light fields are almost ignored [9]. As one of the most important properties, the polarization state of the imaging light wave vector usually contains rich and unique target information about its own radiations and also interaction behaviors with illuminating light beams [10]. Currently, the polarization information of the imaging light waves has been widely used to detect relatively weak radiations out from small or dim targets in complex circumstances, for instance, strong scattering medium [11], functioned material classification, object recognition [12,13] and segmentation [14], and 3D target reconstruction [15,16,17]. In previous research, a dual-polarized light-field imaging approach was proposed for three-dimensional observation [18], whereas the key degree of linear polarization (DoLP) information and the all-in-focus imaging cannot be obtained, which greatly limits the capability and application of target detection and recognition.

In this paper, we demonstrate an integrated plenoptic camera based on an electrically tunable LC device for all-in-focus polarimetric imaging. By using computer numerical control machining and 3D printing, the proposed imaging architecture is integrated into a hand-held prototyped camera, so as to greatly improve the integration of the proposed imaging system and convenience for performing outdoor imaging experiments. Experiments demonstrate that the proposed plenoptic camera can simultaneously realize a dual-mode imaging including the common plenoptic and the polarimetric models without any macroscopic moving parts. With the depth-based rendering, both the all-in-focus images with three polarization states and the all-in-focus DoLP images can be obtained effectively. Thanks to the large depth of field advantage of plenoptic cameras, compared with conventional 2D polarimetric imaging, the rendered all-in-focus imaging approach enables the polarimetric imaging in a larger depth range. In addition, experiments show that the proposed LC structure can effectively enhance the visibility in the scattering medium.

## 2. Materials and Methods

**Figure 1:**
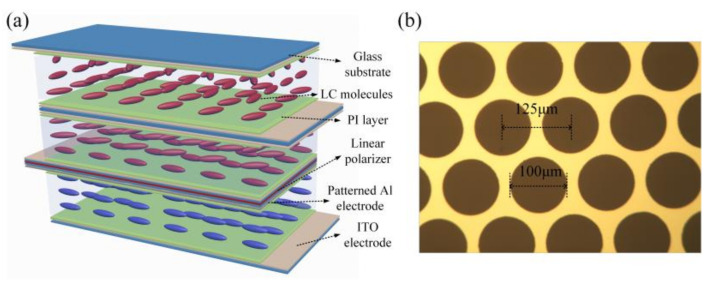
The basic LC architecture for realizing all-in-focus polarimetric imaging. (**a**) The schematic of the hybrid composed of a CTNLC cell and a LCMLA. (**b**) A partial patterned Al electrode of the LCMLA.

As the key functioned component of the proposed plenoptic camera, the detailed schematic of a liquid-crystal microlens array (LCMLA) constructed by a cascaded twist nematic liquid-crystal (CTNLC) cell is depicted in Figure 1. As shown in Figure 1a, the part above a linear polarizer is a CTNLC cell and the part below is a LCMLA. The linear polarizer (USP-50C0.4-38 of OptoSigma, Les Ulis, France) is sandwiched between them. As shown, the CTNLC cell is mainly consisted of two twist nematic LC cells with a total twist angle of 120° (i.e., 60° for each). Two micro-cavities for filling LC materials are formed by stacking three ~500 μm glass substrates. The glass micro-sphere spacers of 10 μm diameter are mixed with the adhesive and then deposited over the surface of the substrates so as to effectively separate three glass substrates leading to both micro-cavities with needed depth mentioned above. The inner side of the top and bottom glass substrates is deposited with a planar indium tin oxide (ITO) electrode of 185 nm thickness, respectively, and the middle glass substrate is also deposited with a planar ITO electrode of 50 nm thickness on both sides, according to common magnetron sputtering. A layer of polyimide film (ZKPI-440 of POME Technology Co., Ltd., Beijing, China) is continuously spin-coated on each planar ITO electrode of the CTNLC cell, and then prebaked for 10 min at 80 °C and cured for 30 min at 230 °C, sequentially. Both prebake and cure operation are performed over a hot plate. The cured PI layer on each planar ITO electrode is then rubbed along a specific direction so as to act as an alignment layer of LC molecules. In each micro-cavity, the rubbing direction shaped over both the top and bottom polyimide layer is differed by 60°, which means a formation of a 60° twist configuration of LC molecules filled later. Although separated by the middle glass substrate, the twist configuration of LC molecules by both micro-cavities already realizes a continuously rotating of 120°, i.e., the total twist angle of CTNLC is 120°. Finally, two layers of long rod-shaped nematic LC materials (E44 of Merck) are fully filled in the formed micro-cavities. Their electro-optical parameters are: n_e_ = 1.7904 and n_o_ = 1.5277 (∆n = 0.2627) and ε_⏊_ = 5.2, ε_//_ =22.0, where ε_⏊_ and ε_//_ are the dielectric constants of LC molecules perpendicular and parallel to the LC director, respectively. The clearing point is ~100 °C and the melting temperature about −6 °C. It is worth mentioning that by using the developed LC mixture, several parameters including a larger LC phase range and a more obvious birefringence difference and a shorter response time can be achieved [19]. The orientation of LC molecules contacted closely with the bottom substrate is set to be parallel to the transmission axis of the linear polarizer used.

The key functional structures of the LCMLA are both ~500 μm glass substrates with different conductive film pre-coated over their inner surfaces. The inner surface of the bottom substrate is deposited with a planar ITO electrode of 185 nm thickness, and that of the top substrate deposited with an aluminum (Al) electrode of 100 nm thickness, according to the common magnetron sputtering. After conventional ultraviolet photolithography and the wet-etching process, the patterned Al electrode with arrayed micro-holes is formed. To improve the utilization efficiency of incident light beams, a patterned micro-hole with a hexagonal period is used to replace the previous quadrilateral period, which means that the filling factor of the formed LCMLA can be increased from ~50.27% to ~58.04%. A partial patterned Al electrode is illustrated in Figure 1b. As it is shown, the diameter of each micro-hole is 100 μm and the center-to-center distance is 125 μm. Similarly, the polyimide layer is also further spin-coated on both the patterned Al and planar ITO electrodes of the LCMLA, and then also prebaked for 10 min at 80 °C and cured for 30 min at 230 °C over a hot plate, sequentially. The cured polyimide layers on the patterned Al and planar ITO electrodes act as an initial alignment layer of LC molecules, which are rubbed anti-paralleled to each other so as to homogeneously align the LC molecules filled later. To satisfy the polarization sensitivity, the initial orientation of LC molecules should be parallel to the transmission axis of the linear polarizer used. Glass microsphere spacers of 20 μm diameter are also mixed with the adhesive and then deposited over a surface of a glass substrate to separate two substrates and shape a micro-cavity. Finally, a layer of LC materials (E44 of Merck) is fully filled into the shaped microcavity to construct the LCMLA architecture.

**Figure 2:**
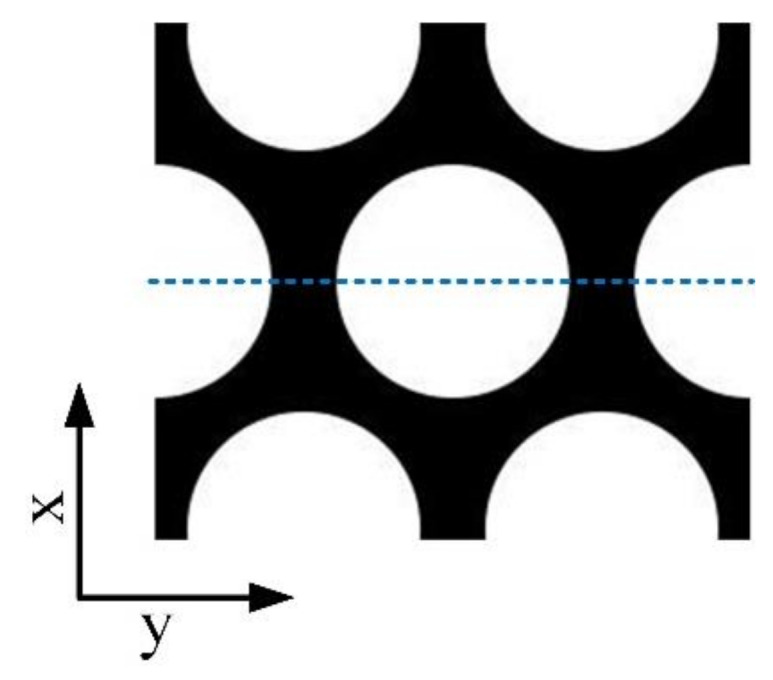
The used simulation electrode pattern.

Based on the common finite-difference method (FDM) [20], we simulate the LC directors in the hexagonal-lattice LCMLA device with MATLAB R2017a. The used simulation electrode pattern is shown in Figure 2. According to previous research experience, the voltage signals with the same value of 4 V are applied over the electrode pattern, and the LC cell thicknesses of 15 μm, 20 μm, and 25 μm, are used so as to effectively select the appropriate thickness needed.

**Figure 3:**
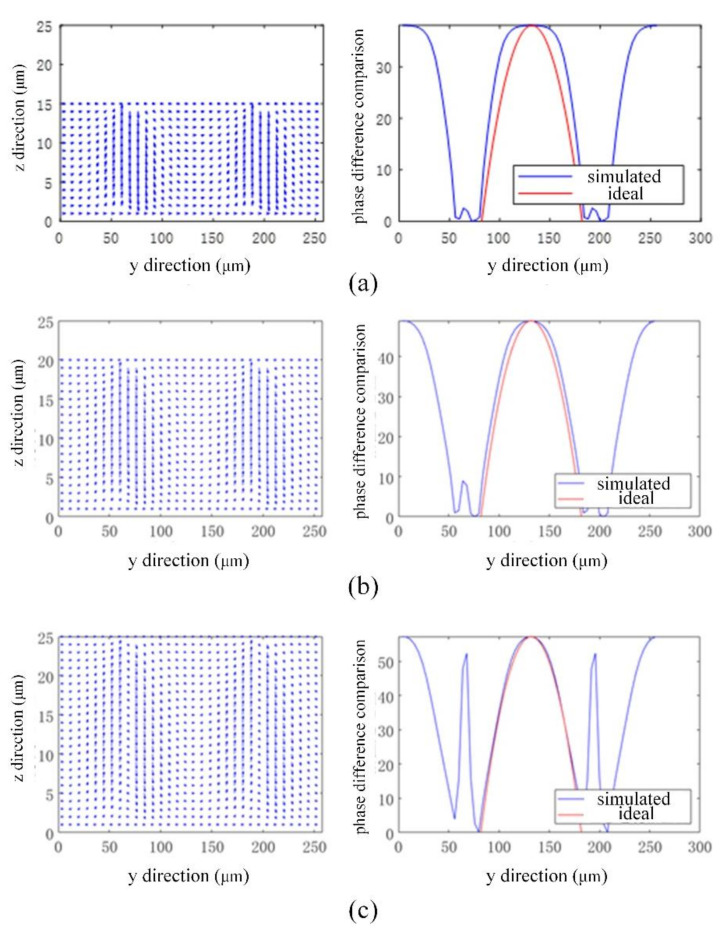
The simulated LC director distribution and the phase retardation difference comparison of (**a**) 15 μm, (**b**) 20 μm, and (**c**) 25 μm.

Figure 3 show the simulated LC director distributions along the blue dotted line in Figure 2, and the phase retardation difference comparison with the ideal profile corresponds to 15 μm, 20 μm, and 25 μm, respectively. As it is shown, when the thickness of the LC cell is 15 μm, the generated phase retardation difference is quite different from the ideal phase retardation difference curve; therefore, its imaging capability is judged to be insufficient. When the thickness of the LC cell is 25 μm, the phase retardation difference in the center is nearly closed to the ideal phase retardation distribution. However, with the increase in the thickness, the LC molecules under the electrode pattern exhibit serious disclination, thus resulting in an uneven phase retardation difference, which will affect the stability of the LC cell. On the other hand, when the thickness of the LC cell is 20 μm, the retardation difference distribution is very closed to the ideal retardation difference, and the disclination phenomenon in an acceptable range compared with that of 25 μm. Therefore, the 20 μm thickness is chosen as the designed thickness. Moreover, according to the simulations, the interaction between holes does not influence the LC director distribution as well as the phase retardation redistribution. Therefore, the designed hexagonal-lattice hole pattern and the cell thickness of 20 μm are determined.

Generally, the Stokes vector ***S*** is a four-element vector which provides quantitative measurements of polarimetric imaging [21]. As a conventional means to display and continuously employing the polarization information of the radiation light waves out from targets in an imaging scene, the degree of polarization (DoP) can be calculated in terms of the Stokes parameters with suitable amplitude or intensity configuration. Among the Stokes parameters, the parameter of *S*_3_ represents the circularly polarized component; the previous research shows that the circularly polarized light has a “polarization memory” characteristic, and thus can be used for enhancing the quality of image recovery [22]. However, in most initiative or passive imaging applications, it is very small and rarely measured. Likewise, there is no effective measurement approach for portable devices. Therefore, *S*_3_ is usually assumed as 0 in this research. Therefore, the DoP and the DoLP can generally be viewed as an equivalent quantity; thus, the Stokes parameters can be further obtained according to a set of radiometric intensity measurements [23] as follows:

(1) $$S=\left[\begin{array}{c} S_{0}\\ S_{1}\\ S_{2}\\ S_{3} \end{array}\right]=\left[\begin{array}{c} \frac{2}{3}(I_{0}+I_{60}+I_{120})\\ \frac{2}{3}(2I_{0}-I_{60}-I_{120})\\ \frac{2}{\sqrt{3}}(I_{60}-I_{120})\\ 0 \end{array}\right]$$

where *S_i_* (*i* = 0, 1, 2, 3) are the Stokes parameters, and *I_θ_* is the intensity of polarized light wave recorded when the linear polarizer in front of the imaging sensors is placed at an angle of *θ* with respect to the *x-*axis. Additionally, the DoLP can be calculated by Stokes parameters as follows:

(2) $$\mathrm{DoLP}=\frac{\sqrt{S_{1}^{2}+S_{2}^{2}}}{S_{0}}.$$

where DoLP is between 0 and 1. For completely linearly polarized light DoLP = 1 and for unpolarized light DoLP = 0. It is worth mentioning that when the circularly polarized component *S*_3_ is considered, due to a relatively long exposure time of image acquisition process, it is equivalent to increase the same value in *I*_0_, *I*_60_, and *I*_120_. According to Equations (1) and (2), the calculated *S*_0_ is larger than actual value, whereas *S*_1_ and *S*_2_ are the same as the actual value due to the subtraction of the calculation. Thus, the calculated DoLP value is less than the actual value for each pixel, with an exception, i.e., when DoLP is 0, the calculated value is the same as the actual value.

**Figure 4:**
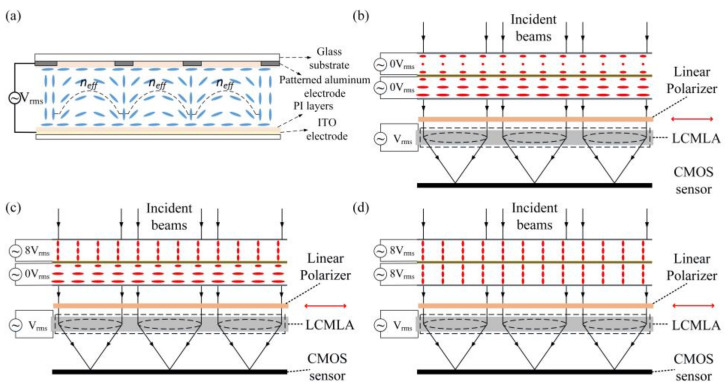
The configuration schematic of the CTNLC cell and the LCMLA coupled with an arrayed CMOS sensor. (**a**) Gradient refractive index profiles corresponding to the extraordinary beams in the LCMLA. (**b**–**d**) Several typical states formed by orderly cascading the CTNLC cell and the LCMLA with featured LC arrangement for recording three different polarizations including *I*_0_, *I*_60_, *I*_120_.

The TNLC cell is an effective broadband polarization rotator which has been widely used in LC displays. The typical milestones are as follows. The operating principle was firstly published by M. Schadt and W. Helfrich [24]. The mathematics concerning the twisted nematics were derived by D.W. Berreman [25]. The operating principle of the planar-aligned positive dielectric nematics was published by M. F. Schiekel and K. Fahrenschon [26]. Those previous studies demonstrate that a beam of specific polarized light wave can be obtained from unpolarized light wave via TNLC/polarizer combination [18,27,28,29]. Under the condition of no external signal voltage being applied or in the off state for each TNLC cell with a 60° twist configuration, the polarization of incident beams are rotated by 60°. When an appropriate external signal voltage is applied, the initial beam rotation disappears. Therefore, three different polarization states such as *I*_0_, *I*_60_, *I*_120_, can be obtained by applying a set of suitable signal voltage. The LCMLA is another key component of the proposed plenoptic camera. Recently, the LCMLA-based plenoptic cameras have attracted a lot of research interests [30,31] owing to several obvious advantages including easy integration with other functioned structure or devices, 2D/3D switchable mode, ease of fabricating [32,33], and the excellent tunability with driving electric field applied [34,35,36,37,38]. In addition, the previously proposed the micrometric LC bubbles do not require the patterned electrode and thus remarkably improve the optical resolution of the LCMLA [39]. In the plenoptic camera proposed by us, the LCMLA is used to form the needed imaging light fields of the targets and scene. The configuration schematic of the CTNLC cell and the LCMLA coupled with an arrayed CMOS sensor is given in Figure 4. As shown in Figure 4a, a gradient refractive index distribution corresponding to the extraordinary beams can be formed so as to result in an effective beam converging towards the focal point, when an appropriate signal voltage is applied on the LCMLA. Generally, the focal length can be easily adjusted by tuning the root mean square (RMS) value of the signal voltage applied. The black dash line indicates a typical equivalent reflective index distribution profile. Several typical states formed by orderly cascading the CTNLC cell and the LCMLA with featured LC arrangement for recording three different polarizations including *I*_0_, *I*_60_, *I*_120_, are shown in Figure 4b–d. The direction of small red arrows represents the light wave transmission direction of the linear polarizer.

For the proposed plenoptic camera, the imaging response time of switching polarization state mentioned above mainly depends on an effective period of reconstructing a stable LC distribution in each TNLC component, which is usually tens of milliseconds. Due to the intrinsic swing characteristics of nematic LC molecules, the response time is usually in the order of 100 ms for the LCMLA with a LC layer of several tens of micron thickness [40].

## 3. Results

### 3.1. Common Optical Properties of the CTNLC and LCMLA

**Figure 5:**
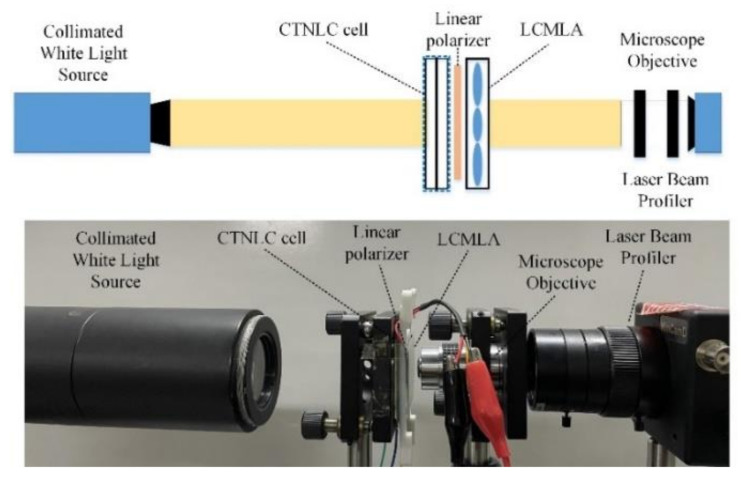
The common testing configuration for measuring the basic parameters of the PSF and the focal length of the LCMLA with the CTNLC cell. The top subfigure shows the measurement principle, and the bottom demonstrates the main experimental set-ups.

To quantitively evaluate the performance of the key CTNLC cell and the LCMLA constructed, two basic parameters of the point spread function (PSF) and the focal length of the developed devices are measured according to a common measurement system, as shown in Figure 5.

As demonstrated, a beam of collimated white beams continuously passes through the CTNLC cell and then the linear polarizer and the final LCMLA. Additionally, the transmitted light fields are then remarkably amplified by a microscope objective of ×40 and 0.65 numerical aperture, and thus captured by a Laser Beam Profiler (WinCamD of DataRay, Inc., Redding, CA, USA). To finely locate the focal planes shaped, the distance between the LCMLA and the microscope objective should be adjusted precisely during experiments. According to our experimental configuration, a relatively accurate focal length should be equal to a sum of the thickness of the glass substrate and the distance between the beam exiting end of the LCMLA and the incident surface of the microscope, whereas each TNLC cell should be adjusted to “on state” by applying a signal voltage of ~8.0 V_rms_. During experiments, the exact values of three polarization components of *I*_0_ and *I*_60_ and *I*_120_ of incident light waves are measured by applying different signal voltage upon the CTNLC cell.

**Figure 6:**
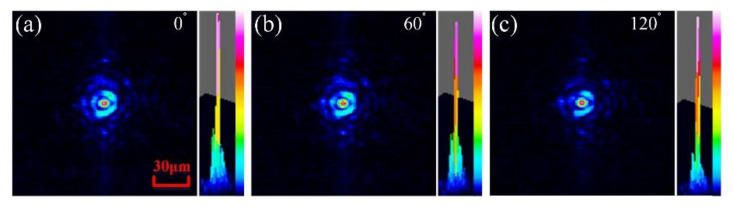
The 2D and 3D PSFs corresponding to different polarization component of *I*_0_ in (**a**) and *I*_60_ in (**b**) and *I*_120_ in (**c**) when a signal voltage of ~3.50 V_rms_ is applied over the LCMLA.

The typical 2D and 3D PSFs corresponding to three polarization components of *I*_0_ in Figure 6a and *I*_60_ in Figure 6b and *I*_120_ in Figure 6c of the LCMLA applied by a signal voltage of ~3.50 V_rms_ are shown in Figure 6. As demonstrated, the shaped focal spots are relatively sharp and the stary light almost disappeared compared with that of the conventional LCMLA without any TNLC structures. The measured focal length is ~1.60 mm. It should be noted that the refractive index of the LC materials utilized in the proposed LC device cannot be perfect axisymmetric parabolic-like. The factors that the phenomenon of a relatively obvious LC molecule dispersion existing will result in an imaging chromatic aberration, and also a relatively large curvature of the refractive index profile shaped in LC layer will introduce relatively strong spherical aberrations [41,42]. According to the measurements, the full width at half maxima (FWHM) of the focal spots is between ~4 μm and ~5 μm, which can be continuously decreased through further optimizing the structural design and the fabrication technology. It can be seen that the developed LCMLA can be effectively used to converge incident beams based on three polarization components including *I*_0_, *I*_60_, *I*_120_, respectively, as shown by Figure 6a–c.

**Figure 7:**
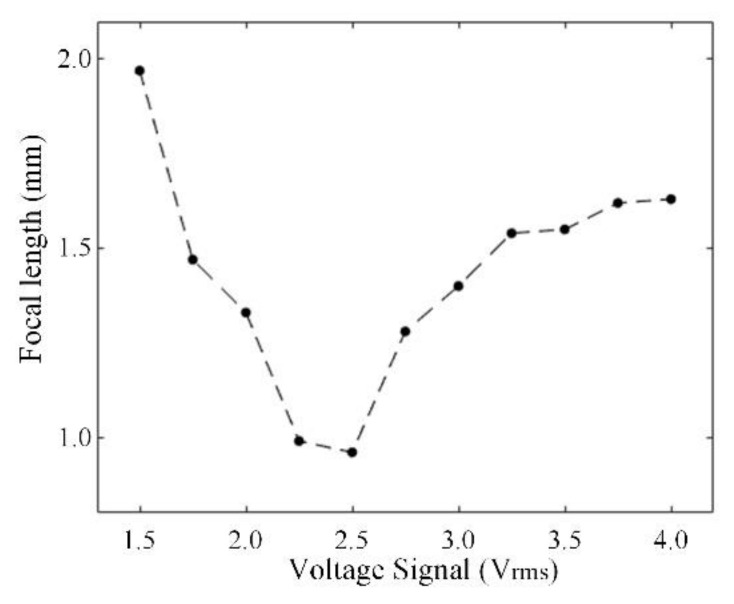
Relationship between the focal length of the LCMLA and the RMS value of the signal voltage applied over it.

The relationship between the focal length of the LCMLA and the RMS value of the signal voltage applied over the LC device is demonstrated in Figure 7. According to our experiments, the current LCMLA can be driven normally in the voltage range from ~1.5 V_rms_ to ~4.0 V_rms_, which is a very favorable factor for constructing the electrical driving and the adjusting system. The realized focal length is in a range from ~0.96 mm to ~1.97 mm, which can be tuned easily so as to present an excellent tunability under the condition of a low signal voltage level. In general, the focal length dependence on the applied signal voltage of a LC microlens exhibits a non-monotonic behavior, that is, it will firstly decrease and then increase with increasing the voltage signals applied. The reasons are already explained in detail in previous research [43].

### 3.2. Typical Imaging Application

**Figure 8:**
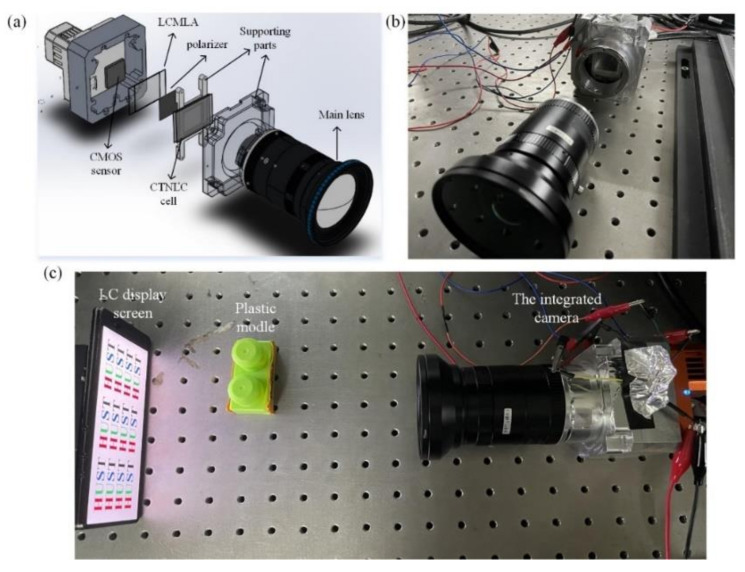
Typical characters of the prototyped camera proposed by us. (**a**) The schematic diagram of the key functioned structure configuration of the camera. (**b**) The optical arrangement of the prototyped camera. (**c**) Imaging experiment arrangement.

Figure 8 demonstrates the typical characters of the proposed prototyped camera. The schematic diagram of the prototyped camera with needed structural configuration is shown in Figure 8a. The main lens and the imaging optical aperture of the proposed camera are shown in Figure 8b. The imaging measurements scene is illustrated in Figure 8c. As shown, the proposed plenoptic camera consists of a CMOS sensor (IMX342 of Sony, Tokyo, Japan) and a CTNLC cell and a LCMLA. A main lens of LF2528M-F with a focal length of 25 mm is used to collect the target radiations. By using computer numerical control machining and 3D printing technology, the above components are fabricated and integrated into a hand-held prototyped camera. The resolution of the CMOS sensor array is 6464 × 4852 and its pixel pitch is 3.45 μm. Both the dynamic range and the signal-to-noise ratio (SNR) are 73 dB and 40 dB, respectively, so as to enable high contrast images. The distance between LCMLA and the CMOS sensor is set as ~1.1mm. The F-number of the main lens is set to match the LCMLA so as to insure a relatively full use of the resolution of the CMOS sensor and further avoid the crosstalk between sub-images. To demonstrate the polarimetric imaging application, a plastic model car and a LC display screen of 5.99 inch are selected as targets. The model car and the LC display screen are placed 100 mm and 210 mm away from the prototyped camera constructed, respectively.

**Figure 9:**
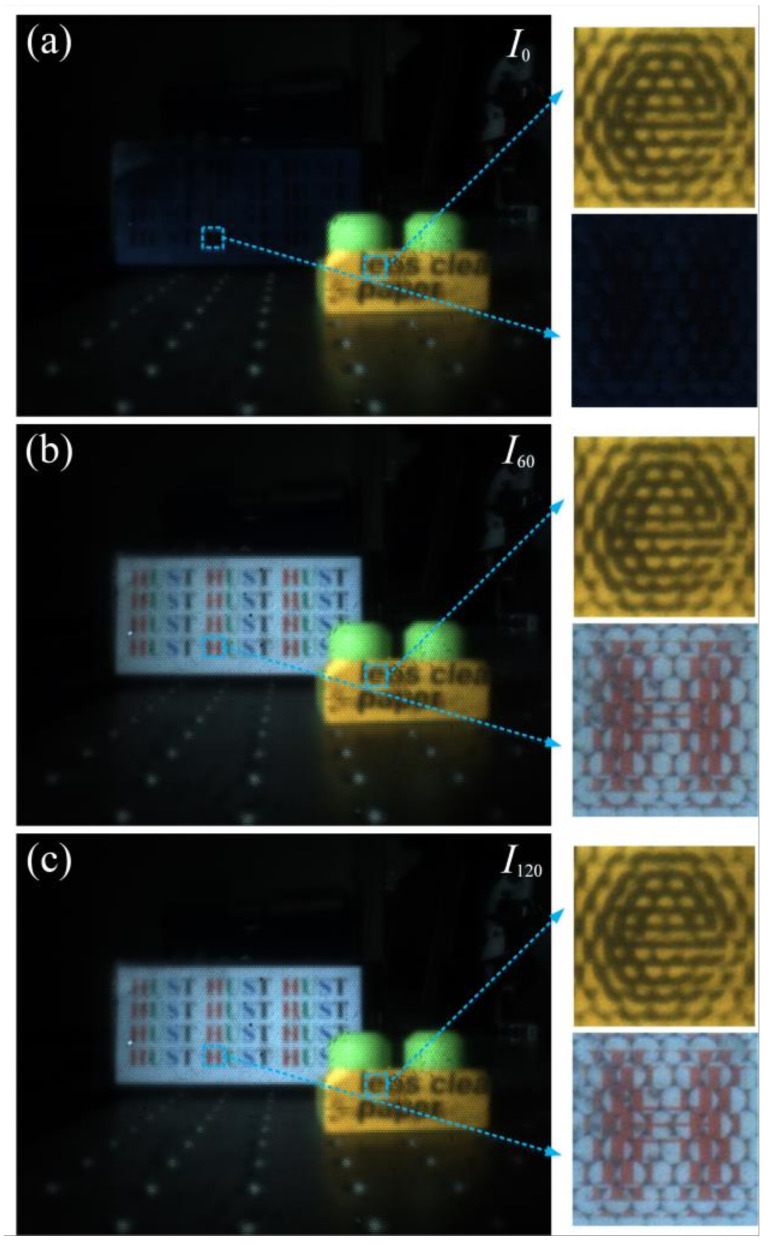
Several typical raw light field images with different polarization components of (**a**) *I*_0_ and (**b**) *I*_60_ and (**c**) *I*_120_ using the prototyped camera applied by a signal voltage of ~3.5 V_rms_ over the LCMLA integrated in the imaging architecture developed.

The raw light field images with three polarization states of *I*_0_ and *I*_60_ and *I*_120_ captured by the prototyped camera developed by us are respectively shown in Figure 9a–c. Both partial enlarged images of the model car and the LC display screen are demonstrated on the right in each subfigure. The pixel resolution of the enlarged part of two letters “e” and “H” are, 237 × 236 and 243 × 253, respectively. Each TNLC cell is turned to “on state” by applying a signal voltage of ~8 V_rms_, and that of applied on the LCMLA is ~3.5 V_rms_. The polarization direction of the linearly polarized light emitted by the LC display screen is 90°, and its components in both the 60° and 120° directions are almost the same; therefore, there is no obvious difference between Figure 9b,c. This can also be observed from Figure 9a, where the position of the LC display screen is almost black.

**Figure 10:**
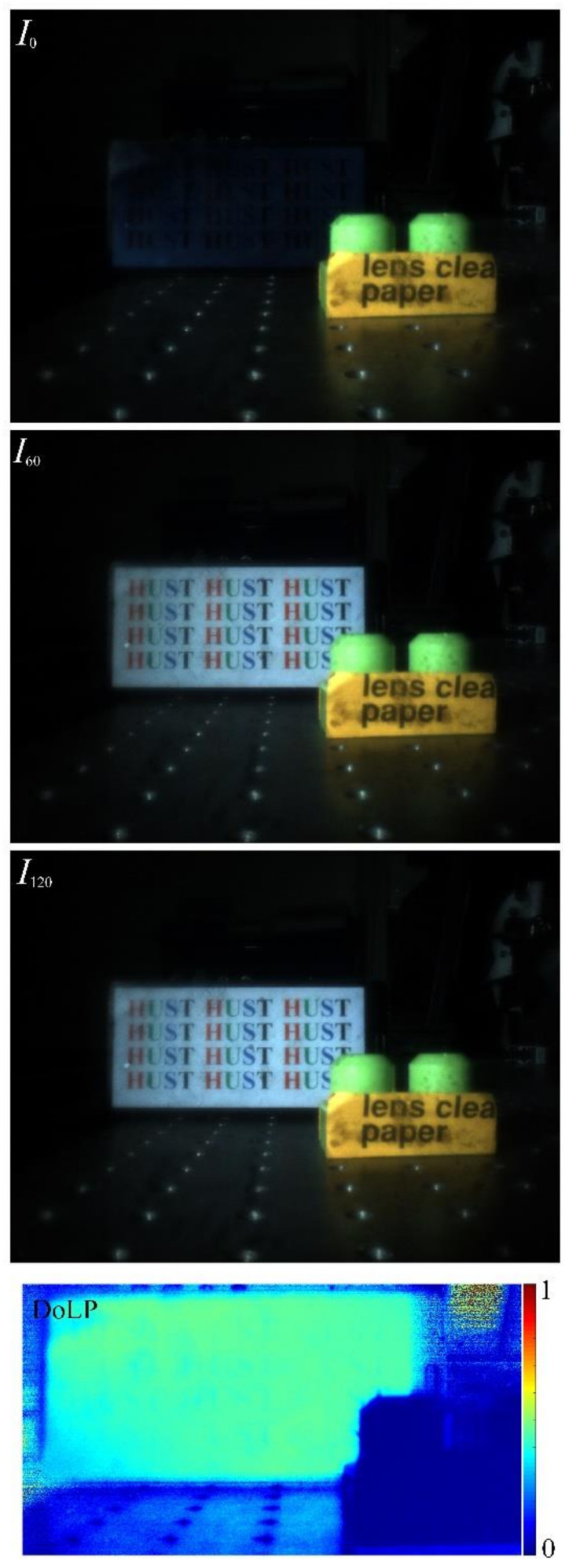
The all-in-focus rendering images of the plastic model car and the LC display screen with different polarization component involving *I*_0_ and *I*_60_ and *I*_120_, as well as a partial all-in-focus normalized DoLP image.

As shown, the light intensity change of the plastic model car is not obvious in different polarization states, whereas an obvious variance of the text and the picture on the LC display screen can be viewed due to the significant difference in polarization characteristics. As it is known, the plenoptic cameras will present a larger depth of field compared with that of conventional 2D cameras. By further using digital re-focusing operation, different imaging plane corresponding to that in objective space can be rendered “in focus” according to different patch size selected. However, only the selected plane is refocused “in focus” with a specific patch size, and then the artifacts will be produced on other planes, especially on the planes far apart. When the obtained images are used to perform particular functions such as target detection or recognition, the produced artifacts are undesired. In a multi-depth target environment, the method of manually setting the patch size is no longer feasible. According to the depth-based rendering method, the all-in-focus images can be obtained, thereby achieving a tunable polarimetric imaging in a relatively large depth range. Based on the sum of an absolute difference algorithm, a 7 × 7 block is used for image matching to obtain disparity information, thereby achieving the depth-based all-in-focus rendering. The detailed depth-based rendering process refers to [7] (p. 7). The all-in-focus rendering images of both the plastic model car and the LC display screen with different polarization component including *I*_0_ and *I*_60_ and *I*_120_, as well as the partial all-in-focus DoLP image, are shown in Figure 10. As demonstrated, the objects in a specific depth range are already rendered in-focus via the depth-based rendering approach. The plastic model car and the LC display screen can be easily identified due to their significant polarization difference.

**Figure 11:**
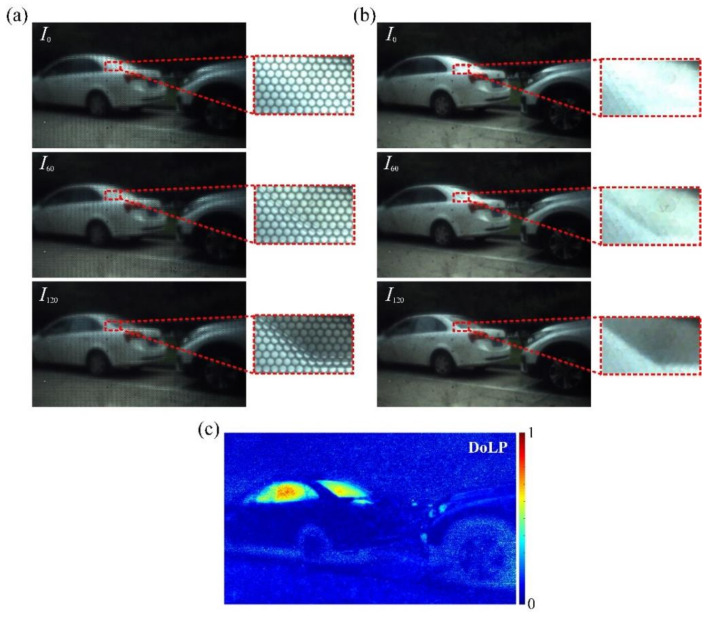
The typical outdoor imaging characters. (**a**) The captured raw light field images and (**b**) the rendered all-in-focus images with three polarization orientations mentioned. (**c**) The calculated all-in-focus normalized DoLP image.

Since the proposed prototyped camera has been integrated into a held-hold imaging system, a typical outdoor polarized plenoptic imaging experiment is conducted, as shown in Figure 11. The captured raw light field images and the rendered all-in-focus images with needed polarization state including *I*_0_ and *I*_60_ and *I*_120_ are illustrated in Figure 9b and Figure 11a, respectively. Each of the small images surrounded by a red dashed frame are enlarged so as to clearly demonstrate an obvious brightness difference of the window materials and the car body, which can be apparently viewed in each polarized state mentioned. The calculated all-in-focus DoLP image is shown in Figure 11c. For the captured raw light field images in Figure 11a, the pixel resolution of the enlarged parts is 420 × 265, and that of the rendered all-in-focus images shown in Figure 11b is 112 × 67, which thus indicates a loss of spatial resolution due to the refocusing process.

**Figure 12:**
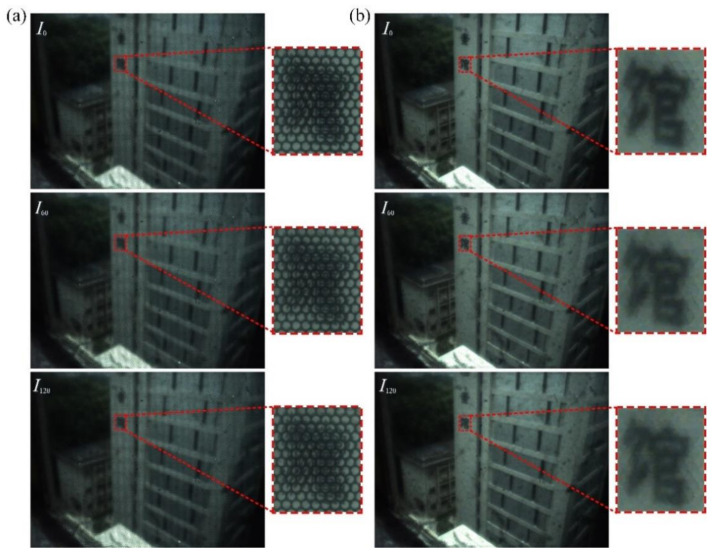
Typical outdoor imaging measurements according to our imaging architecture using a polarization system of {*I*_0_, *I*_60_, *I*_120_}. (**a**) The raw light field images and (**b**) the rendered all-in-focus images.

**Figure 13:**
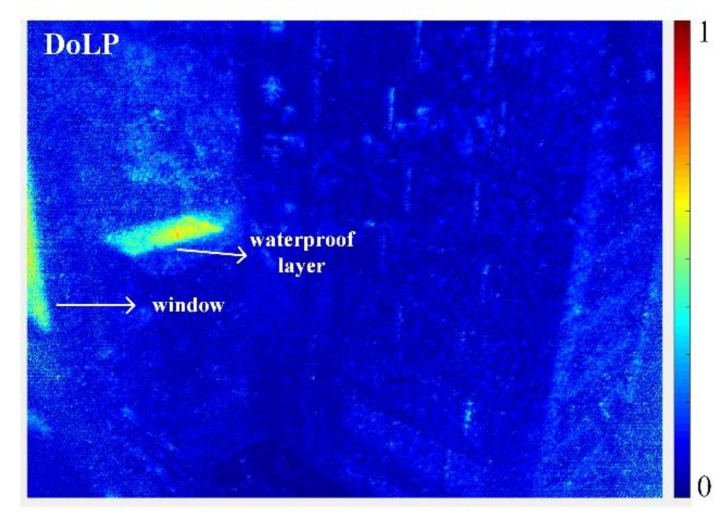
The calculated all-in-focus DoLP image.

As shown, both the side and rear windows of the car are strongly exhibited by the all-in-focus DoLP images. When its windows are selected as a target, the related images with different polarization states including *I*_0_ and *I*_60_ and *I*_120_, can be captured by the proposed imaging architecture. Through electrically tuning the polarization state mentioned, the strongly reflected sunlight of the windows can be effectively suppressed, so as to outstand their morphology and outline characters for easily performing graphic information processing. Another typical outdoor imaging measurement is shown in Figure 12. The captured raw light field images and the rendered all-in-focus images with three polarization states are illustrated in Figure 12a,b, respectively. For the captured raw light field images in Figure 12a, the pixel resolution of the enlarged parts is 334 × 401, and that of the rendered all-in-focus images shown in Figure 12b 84 × 100, which also indicates the loss of spatial resolution due to the refocusing process. The calculated normalized all-in-focus DoLP image is demonstrated in Figure 13.

As shown, the polarization images of the waterproof layer show obvious brightness differences. The window and waterproof layer exhibit obvious polarization characters in the all-in-focus DoLP image, which is consistent with the theoretical prediction. Compared with conventional plenoptic cameras only capturing the geometric aspects of the plenoptic function, the proposed imaging architecture or prototyped polarimetric plenoptic camera can be more effectively utilized to detect the particular objects with polarization information, which means an effective improvement of the detection and recognition efficiency of the plenoptic imaging system.

### 3.3. Visibility Enhancement in Scattering Medium

**Figure 14:**
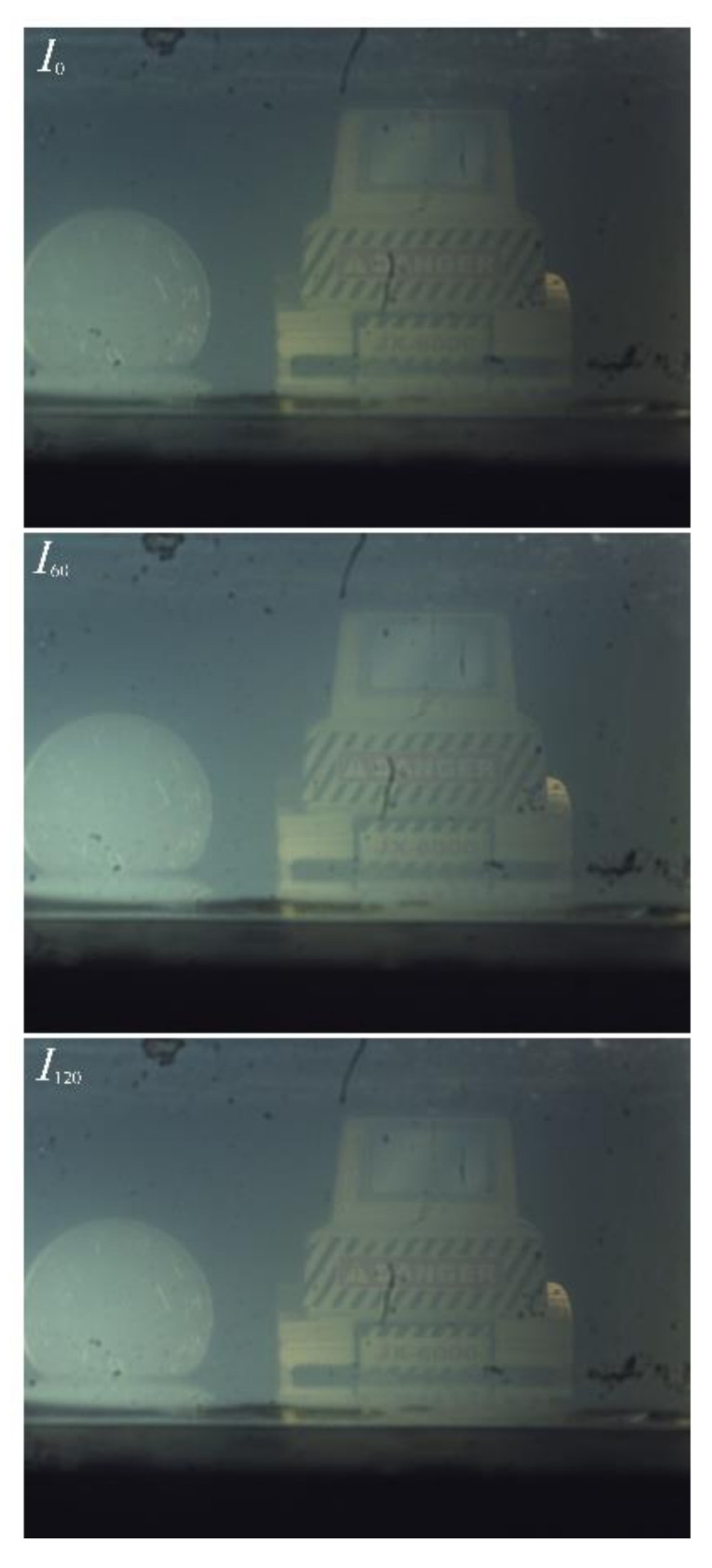
Three 2D light intensity images captured according to three polarization states, including *I*_0_ and *I*_60_ and *I*_120_, respectively.

In the scattering medium, both the sharpness and the contrast of the optical images usually suffer degradations, as shown in several typical situations such as conducting imaging detection through haze, or performing underwater imaging [44,45,46]. Many previous studies revealed that the imaging quality in the scattering medium can be effectively improved by polarization information. In this section, two different polarization-based methods are employed for remarkably enhancing the imaging quality in the scattering medium. A typical scattering medium circumstance is constructed by adding five drops of whole milk to a 10 cm × 5 cm × 6 cm water tank full of pour water. The constructed water tank is placed on an optical table and a LED photography light that emits diffuse white light is used as the illuminating source to simulate natural light. A coin and a yellow model dozer are placed behind the water tank as targets; nothing is placed elsewhere in the field of view. During measurements, the LCMLA is removed for obtaining an imaging effect with more intuitive vision and also avoiding the block effects as a kind of imaging noise introduced by re-focusing operation. Similar to the experiments in above section, 2D light intensity images with three polarization states of *I*_0_ and *I*_60_ and *I*_120_ are captured by the proposed LC-based imaging system, as shown in Figure 14. As shown, the image in the water tank region exhibits visual whitening effect, which is caused by the backscattered light of the constructed scattering medium. In fact, the backscattered light is the most important factor causing image degradation in scattering media, generally.

**Figure 15:**
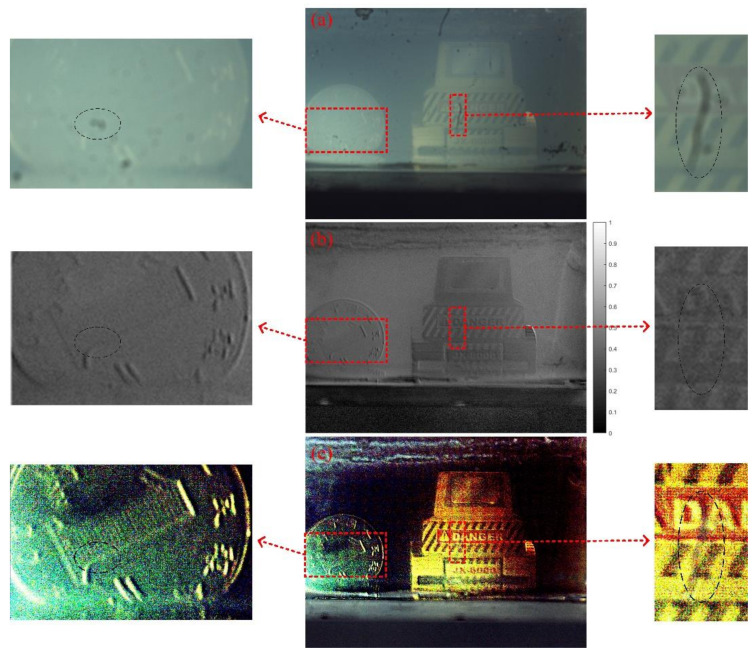
The typical cases about the visibility enhancement in scattering medium. (**a**) Imaging character under the polarization state of *I*_60_. (**b**) The calculated grayscale DoLP image. (**c**) The recovered image according to polarization difference information. Partial enlarged images are also given.

The visibility enhancement in scattering medium is realized through two methods basically, as shown in Figure 15. For comparison, the imaging characters corresponding to the polarization state of *I*_60_ are also shown in Figure 15a. The first method is to restore target features by calculating the grayscale DoLP, as shown in Figure 15b. The second method is to remove the visual effects of the scattering medium by the polarization-difference (PD) information. In the experiments, two patterns with typical polarization states of *I*_60_ and *I*_0_ are selected as input images. The PD-based recovered image is illustrated in Figure 15c, the detailed process of which can refer to Ref. [44].

Several partially enlarged sub-images are also located at the left and right sides, respectively. For Figure 15, the enlarged sub-images at the left and right sides are enlarged 3.06 times and 4.04 times, respectively. In these enlarged areas, both the grayscale DoLP image and the recovered image based on the polarization difference exhibit clearer texture features of the local targets. In particular, the recovered image according to the polarization difference presents a better image contrast and visibility. Moreover, some dot-shaped and short linear-shaped pieces of dirt can be seen in Figure 15a, and two typical dust areas are circled by black dotted lines, where the CMOS sensors are already contaminated by them. By carefully comparing the featured areas with small pieces of dust, both the DoLP image and the PD-based recovered image demonstrate a certain inhibitory action to that contamination noise, because their intensity is greatly reduced. The reason can be attributed to the subtraction operation performed in the calculation process. Experiments demonstrate that both the grayscale DoLP image and the PD-based recovered image present clearer target features than that of the original 2D light intensity image, and the PD-based recovered image has a better image contrast and visual visibility, so as to significantly enhance the imaging quality in the scattering medium. Therefore, the proposed LC device is expected to have excellent application potential in scattering medium imaging.

## 4. Conclusions

In this paper, an integrated plenoptic camera based on a key electrically tunable LC device for realizing all-in-focus polarimetric imaging is proposed. By using computer numerical control machining and 3D printing, the proposed polarimetric imaging architecture can be integrated into a hand-held prototyped camera, so as to greatly improve the applicability of the imaging equipment for outdoor imaging measurements. Experiments demonstrate that the proposed imaging approach can simultaneously realize dual-mode (involving: plenoptic and polarimetric) imaging without any macroscopic moving parts. Based on the depth-based rendering method, both the all-in-focus images with needed polarization states and the all-in-focus DoLP images can be effectively obtained. Due to the large depth of field advantage of plenoptic cameras, the proposed camera enables polarimetric imaging in a larger depth range than conventional 2D polarimetric cameras. The raw light field images with three polarization states of *I*_0_ and *I*_60_ and *I*_120_ can be captured by the proposed imaging architecture in a switching time of several tens of milliseconds. Some local patterns selected as interested target features can be effectively suppressed or obviously enhanced by switching the polarization state mentioned. According to experiments, the visibility in the scattering medium can also be obviously enhanced. It can be expected that the proposed imaging approach will play an important role in promoting imaging detection efficiency in complicated circumstances.

## Data Availability

Not applicable.
